# Species-specific differences across the rural–urban spectrum for perceived veterinary visit costs among U.S. pet owners

**DOI:** 10.3389/fvets.2026.1879445

**Published:** 2026-07-01

**Authors:** Clinton L. Neill, Abby ShalekBriski

**Affiliations:** 1 Department of Population Medicine and Diagnostic Sciences, College of Veterinary Medicine, Cornell University, Ithaca, NY, United States; 2 Applied Economics Consulting, LLC, Blacksburg, VA, United States; 3 Oklahoma State University, Stillwater, OK, United States

**Keywords:** companion animals, cost perception, feline, health services access, rural–urban differences, veterinary economics

## Abstract

**Introduction:**

Financial barriers are among the most significant threats to companion animal welfare in the United States, yet little is known about how pet owners’ perceived costs of veterinary care vary by geography or species. Rural cat owners in particular face compounding access challenges, including lower veterinary availability, reduced care-seeking norms, and potential differences in price expectations relative to non-rural counterparts. This study examines whether rural, suburban, and urban pet owners differ in their perceived cost of a routine veterinary visit and whether any such perception gap is species-specific.

**Methods:**

Cross-sectional online survey of US adults administered in November 2025. The sample comprised 2,216 dog- and cat-cost observations from 1,885 unique respondents (574 Rural, 759 Suburban, 552 Urban). Respondents reported their expected cost of a routine veterinary visit for their dog, cat, or both. Perceived costs were log-transformed to address right skew. Ordinary least squares regression models estimated the association of community type (Rural [reference], Suburban, Urban) on log-transformed perceived cost, controlling for household income, age, gender, and established veterinarian status. A species-by-community interaction term tested whether the community-type effect differed between dog and cat owners.

**Results:**

The rural versus non-rural cost perception gap was species-specific. For dogs, perceived visit costs did not differ significantly by community type. For cats, rural owners perceived substantially lower routine visit costs than suburban and urban owners. Those without an established veterinarian also held significantly lower cost expectations.

**Discussion:**

Rural cat owners hold lower perceived routine veterinary visit costs than non-rural counterparts, which could be attributable to reduced care exposure, local fee anchoring, and cultural ownership norms. The absence of a parallel effect among dog owners suggests this pattern reflects something specific to rural feline ownership rather than a general rural information disadvantage. These findings extend the literature on rural veterinary access disparities by identifying a cognitive mechanism that may compound structural barriers and contribute to the rural feline preventive care deficit. Interventions targeting proactive cost communication, pricing transparency, and community outreach programs could improve financial preparedness among rural cat owners and reduce the gap between cost expectations and actual care costs.

## Introduction

According to the American Veterinary Medical Association (AVMA), approximately 57% of US households owned at least one pet by 2020, with an estimated 88.8 million dogs and 61.9 million cats across 62 million and 37 million households, respectively (1). Total US consumer expenditure on pets exceeded $95 billion in 2019, with veterinary services and medications accounting for approximately one-third of that figure (2). Despite this scale of investment, the distribution of veterinary care across the US population is uneven. The structure of the veterinary industry shapes how supply-side constraints translate into disparities in access to care (3). Financial barriers are regarded as among the most significant threats to companion animal welfare in the United States (4). This characterization is consistent with findings from Neal and Greenberg (5), LaVallee et al. (6), and King et al. (7), which document financial barriers as a primary driver of unmet veterinary need at the national level. National survey data indicate that more than one quarter of all pet-owning households reported being unable to afford veterinary care in the preceding 2 years (5).

The geography of veterinary care access has emerged as a prominent thread in the literature, mirroring longstanding rural–urban disparities in human healthcare. Rural populations in the United States face well-documented challenges in accessing health services. These access problems include greater distances to providers, higher rates of uninsurance, lower median household incomes, and more limited availability of specialist care (8, 9). These structural conditions are echoed in the veterinary domain. Rural and remote areas are disproportionately represented among so-called veterinary care deserts. These care deserts are denoted as geographic areas defined by limited availability, affordability, and physical access to veterinary services. A recent national mapping exercise finds that approximately 21 million US households and 25.2 million companion animals reside in counties ranked in the lowest quartile of the Veterinary Care Accessibility Score (5). Prior empirical work confirms a significant spatial income gradient in veterinarian earnings, which increases with local practice density. This spatial gradient creates rural workforce climates that are economically disadvantageous for new graduates (10). These conditions are compounded by veterinary students exhibiting declining interest in rural practice over the course of their training (11, 12). Federal loan repayment programs such as the Veterinary Medicine Loan Repayment Program have sought to address these shortages created by these conditions, though program funding has remained modest relative to need (11).

Within this access landscape, the financial cost of veterinary services occupies a central role. A 2025 national survey of US pet owners commissioned by PetSmart Charities and conducted by Gallup found that 52% of dog and cat owners reported skipping or declining needed veterinary care in the prior year. Of those owners, 71% attribute their decision to financial considerations (13). Among owners who declined care due to cost, 73% were not offered a lower-cost alternative by their provider.

The AVMA’s 2025 Pet Ownership and Demographics Sourcebook reports that the average self-reported cost of the most recent veterinary visit was $200, with dog owners reporting slightly higher costs ($220) than cat owners ($202), consistent with a persistent species-level gap in veterinary expenditure documented across multiple survey cycles (14, 15). However, a critical and underexplored distinction exists between the actual cost of veterinary care and pet owners’ perceived cost. More specifically, what owners believe or expect a visit to cost.

Perceived cost is a proximate determinant of care-seeking behavior (16, 17). For the purposes of this study, perceived cost is the dollar amount a pet owner *believes* a veterinary visit will cost them on their next visit. Owners who underestimate prevailing costs may be unprepared for when care is needed, potentially forgoing or delaying treatment as a result. Those who overestimate may preemptively self-ration care they could in fact afford. Understanding how cost perceptions are distributed across geographic and demographic groups has direct implications for patient care and the economics of veterinary practices.

Despite the importance of perceived cost in healthcare utilization models, relatively little research has examined the demographic and geographic correlates of veterinary cost perception directly. The broader literature on health information access offers a starting point. Rural residents often face systematic disadvantages in health acquisition, with lower access to primary care providers as information sources, less frequent internet use for health information, and lower health literacy more generally (18). Similarly, price knowledge in veterinary medicine is usually acquired through direct experience with care. Therefore, rural pet owners may have fewer opportunities than their urban counterparts to be exposed to price knowledge, as there are fewer veterinary providers per household. Consequently, rural pet owners may have different cost expectations than their more urban counterparts. From another perspective, rural practices may offer lower prices due to lower overhead costs and reduced demand. This type of pricing structure may further anchor rural owners’ cost expectations below prevailing urban fee schedules.

Cats consistently receive less veterinary care than dogs across every measure tracked by the AVMA. Cats are shown to visit the veterinarian approximately 0.7 times per year compared to 1.5 times for dogs (14). In 2024, 28% of cat-owning households reported spending nothing on veterinary care in the preceding year compared to 16% of dog-owning households (14). The dog-cat gap in utilization has been attributed to a set of complex factors, including cats’ ability to mask illness, the perceived difficulty of transporting cats, culturally lower expectations of feline veterinary engagement, and casual acquisition (19, 20). The rural–urban dimension may interact with these species-level patterns in ways that have not previously been examined.

The existing literature on access to veterinary care has made substantial progress in characterizing financial and geographic barriers, identifying high-need communities, and documenting the consequences of unmet need for animal welfare (6, 21, 22). Several studies have examined how demographic characteristics such as race, ethnicity, income, and financial fragility predict pet owners’ perceived access to care (7, 23). However, the specific question of how pet owners’ perceptions of the cost of veterinary services vary by community type (urban, suburban, rural), and whether this variation differs between dog and cat owners, has received no empirical attention. If rural pet owners underestimate the cost of routine care, they may be unprepared for the financial demands of acute or emergency treatment. If so, this perception is an information problem that would be responsive to cost-based messaging or transparent pricing interventions such as posted prices in clinic or online. Conversely, cost barriers may reflect affordability constraints rather than being unaware of price information if rural owners’ expectations are well-calibrated to their local fee environment.

The study addresses this gap using data from a national online survey of US pet owners conducted in November 2025. We estimate OLS regression models examining the association between community type and perceived routine veterinary visit costs, and formally test whether that association differs between dog and cat owners. The findings carry implications for veterinary pricing communication, rural outreach program design, and understanding the behavioral mechanisms underlying rural disparities in companion animal preventive care.

## Methods

Data were drawn from a cross-sectional online survey administered to a national online sample of US adults in November 2025. The survey examined consumer preferences and spending behaviors related to pet ownership and veterinary service utilization. Participants were recruited through a panel provider and screened for consent prior to participation. The panel employed quota sampling targeting age, gender, and geographic region to approximate U.S. adult demographics, based on the U.S. Census benchmarks across these dimensions. The overall survey completion rate among those who began the survey was approximately 68%. The study was conducted in accordance with standard ethical requirements for non-identifiable survey research and approved by Cornell University’s Institutional Review Board.

The analysis focused on respondents’ perceptions of the cost of a routine veterinary visit, veterinary access characteristics, and related behaviors. Respondents who owned dogs were asked to estimate the cost of a routine visit for their dog; cat owners were asked the equivalent question for their cat. Respondents owning both species answered both questions.

### Sample

The full survey yielded 3,009 completed responses across pet and non-pet owners. The sample is restricted to pet owners who provided a cost estimate for at least one species (dog or cat) and reported a current community type (Rural, Suburban, or Urban). Respondents entered their perceived cost of a routine veterinary visit for their dog and/or cat in separate open-ended numeric fields. The exact survey wording was: “How much do you think a routine veterinary visit (e.g., a standard wellness exam) would cost for your [dog/cat]? Please enter your best estimate in dollars.” Responses were cleaned prior to analysis: non-numeric entries (e.g., “do not know,” “free”) were coded as missing; range responses (e.g., $100–$150) were replaced with the midpoint; and entries with minor formatting issues (e.g., “$150.00,” “150 dollars”) were parsed to numeric values. A floor of $5 was applied to remove implausible entries—specifically, entries below $5 were excluded as likely data-entry errors given that no routine veterinary encounter in the current US market is plausibly priced below this threshold—, and an upper ceiling was set at the 99th percentile of the distribution for each species separately ($800 for dogs, $700 for cats) to limit the influence of extreme outliers (applied separately by species to preserve distributional symmetry given meaningfully different cost ranges for dogs and cats). A total of 31 respondents entered “free,” “$0,” or equivalent; these were coded as missing because the question asked for a dollar estimate of expected out-of-pocket cost and a zero response could not be unambiguously reconciled with ownership of a paid wellness plan versus genuine zero-cost expectation. Recoding these as $1 (to permit log transformation) did not materially alter any primary estimates. The results are also robust to alternative trimming specifications (e.g., 95th-percentile ceiling, no floor). After cleaning, the dataset comprised 1,245 dog-cost observations and 971 cat-cost observations, for a total of *N* = 2,216 observations from 1,885 unique respondents. Community type was self-reported, with 574 respondents identifying as Rural, 759 as Suburban, and 552 as Urban.

### Statistical analysis

Respondent characteristics and perceived visit costs were summarized by community type using means and standard deviations for continuous variables and percentages for categorical variables. Group differences in continuous characteristics were assessed using Kruskal–Wallis tests; differences in categorical characteristics were assessed using Pearson chi-square tests.

Raw cost distributions were right-skewed (Shapiro–Wilk *p* < 0.001), consistent with typical healthcare cost data, and perceived costs were natural log-transformed prior to all inferential analyses. Four covariates were included in the regression models: (1) household income, calculated from the midpoint of each reported income category and expressed in $10,000 units; (2) respondent age in years; (3) gender (Man [reference], Woman, Other/Prefer not to say); and (4) a binary indicator of whether the respondent had an established veterinarian (Yes [reference] vs. No). Covariate selection followed a strategy guided by both theoretical relevance and empirical stability. Additional candidate variables including education, race and ethnicity, and number of pets in the household, were evaluated during model development but introduced substantial multicollinearity (variance inflation factors consistently exceeding conventional thresholds), inflating standard errors and destabilizing coefficient estimates. The final covariate set represents the theoretically grounded, empirically stable specification that maximizes interpretability without inducing collinearity. Two nested OLS models were estimated with log-transformed perceived cost as the outcome:

Model 1 (main effects): log(cost) ~ community + species + income + age + gender + has_vet;
Model 2 (interaction): log(cost) ~ community × species + income + age + gender + has_vet.

To account for within-person correlation arising from respondents who provided cost estimates for both species, standard errors were clustered at the respondent level. The joint significance of the species × community interaction terms was assessed via a Wald test comparing Models 1 and 2. Estimated marginal means by species and community type were derived from Model 2 with covariates held at their observed distributions, and pairwise Rural versus Urban contrasts within each species were computed with Bonferroni correction. All analyses were conducted in R (version 4.3.0). Statistical significance was assessed at *α* = 0.05. OLS with log-transformation was preferred over generalized linear model alternatives because the log-transformation produced approximately normally distributed residuals, the specification provides directly interpretable proportional effects, and cluster-robust standard errors address residual heteroskedasticity. Breusch-Pagan tests confirmed heteroskedasticity in the untransformed models, supporting the use of robust standard errors in the log-transformed specification.

## Results

The sample included 2,216 cost observations from 1,885 unique respondents, distributed across community types as follows: 574 Rural (30.5%), 759 Suburban (40.3%), and 552 Urban (29.3%). Sample characteristics by community type are presented in Table 1. Rural respondents were slightly older on average (mean 46.2 years, SD = 15.4) than Suburban (44.8 years, SD = 14.9) and Urban (43.1 years, SD = 15.1) respondents (*p* = 0.045). Rural respondents also reported lower median household incomes ($32,500) than Suburban ($37,500) and Urban ($42,500) respondents (*p* = 0.021). Gender composition did not differ significantly across community types (Female: 54.4% Rural, 53.8% Suburban, 55.1% Urban; *p* = 0.950). Rural respondents were more likely to have grown up in a rural area (62.1%) than Suburban (53.4%) or Urban (41.7%) respondents (*p* < 0.001). The majority of respondents across all community types reported having an established veterinarian (Rural: 87.3%; Suburban: 89.1%; Urban: 85.6%; *p* = 0.810). Dog ownership was most prevalent among Rural respondents (57.6%) and cat ownership was slightly higher among Urban respondents (54.9%), though neither difference was statistically significant (*p* = 0.435 and *p* = 0.358, respectively).

**Table 1:** Respondent characteristics and perceived routine veterinary visit costs by community type (*n* = 1,885).

Respondent characteristics	Rural (*n* = 574)	Suburban (*n* = 759)	Urban (*n* = 552)	*p* value
Age, mean (SD), years	46.2 (15.4)	44.8 (14.9)	43.1 (15.1)	0.045^a^
Household income, median, $	32,500	37,500	42,500	0.021^a^
Female, %	54.4	53.8	55.1	0.950^b^
Grew up in rural area, %	62.1	53.4	41.7	<0.001^b^
Has established veterinarian, %	87.3	89.1	85.6	0.810^b^
Dog owner, %	57.6	56.8	52.4	0.435^b^
Cat owner, %	49.1	50.3	54.9	0.358^b^
Perceived dog visit cost, $				
Mean (SD)	197.79 (242.73)	193.77 (230.49)	243.17 (326.32)	0.205^a^
Median	125.00	125.00	150.00	
Perceived cat visit cost, $				
Mean (SD)	143.60 (178.61)	174.79 (240.92)	204.31 (262.64)	0.003^a^
Median	100.00	100.00	120.00	

a Kruskal–Wallis test.

b Pearson chi-square test.

Perceived cost values reflect cleaned, outlier-trimmed estimates (floor = $5; ceiling = 99th percentile: $800 for dogs, $700 for cats). SD, standard deviation.

### Descriptive statistics

Perceived routine veterinary visit costs were right-skewed across both species and all community types (Shapiro–Wilk *p* < 0.001), and are log-transformed for regression. For dogs, mean perceived costs were similar across community types: $197.79 (SD = $242.73) among Rural, $193.77 (SD = $230.49) among Suburban, and $243.17 (SD = $326.32) among Urban respondents. Median perceived dog visit costs were $125.00 for Rural and Suburban respondents and $150.00 for Urban respondents. For cats, a gradient was more apparent, with means of $143.60 (SD = $178.61) among Rural, $174.79 (SD = $240.92) among Suburban, and $204.31 (SD = $262.64) among Urban respondents, with medians of $100.00, $100.00, and $120.00, respectively.

### Regression analysis: community type, species, and their interaction

Table 2 presents results from the two nested OLS regression models with log-transformed perceived visit cost as the outcome (*N* = 2,216 observations from 1,885 unique respondents; cluster-robust standard errors). In the main-effects model (Model 1), Urban community type was associated with significantly higher perceived visit costs relative to Rural areas (*β* = 0.143, SE = 0.060, *p* = 0.017), corresponding to approximately 15.4% higher perceived cost (95% CI: 2.6, 29.7%). Suburban community type did not differ significantly from Rural (*β* = 0.049, SE = 0.050, *p* = 0.332). Cat-cost observations were associated with significantly lower perceived visit costs than dog-cost observations (*β* = −0.183, SE = 0.027, *p* < 0.001), corresponding to approximately 16.7% lower perceived cost for cats relative to dogs.

**Table 2:** Log-OLS regression models of perceived routine veterinary visit cost.

	Model 1 (main effects)		Model 2 (interaction)	
Coefficient	β (SE)	95% CI	β (SE)	95% CI
Community type (ref: rural)				
Suburban	0.049 (0.050)	[−0.050, 0.148]	−0.019 (0.057)	[−0.131, 0.093]
Urban	0.143* (0.060)	[0.026, 0.260]	0.081 (0.067)	[−0.049, 0.212]
Species (ref: dog)				
Cat	−0.183* (0.027)	[−0.237, −0.129]	−0.285* (0.043)	[−0.369, −0.200]
Community × species interactions				
Suburban × cat	–	–	0.155* (0.064)	[0.030, 0.280]
Urban × cat	–	–	0.139* (0.068)	[0.006, 0.272]
Demographics				
Income (per $10,000)	0.009† (0.005)	[−0.001, 0.019]	0.009 (0.005)	[−0.001, 0.019]
Age (years)	−0.007* (0.001)	[−0.010, −0.005]	−0.007* (0.001)	[−0.010, −0.005]
Gender: woman	0.074 (0.045)	[−0.014, 0.161]	0.074 (0.045)	[−0.013, 0.162]
No established veterinarian	−0.231* (0.086)	[−0.400, −0.063]	−0.231* (0.086)	[−0.399, −0.062]
*N*	2,216		2,216	
*R* ^2^	0.044		0.045	
Adjusted *R*^2^	0.040		0.041	

Coefficients are log-scale estimates; proportional differences can be obtained as exp(β) − 1. Standard errors are cluster-robust (HC3 estimator) clustered at the respondent level to account for within-person correlation in the pooled dog/cat dataset. Reference categories: community type = rural; species = dog; gender = man; established veterinarian = yes. *p ≤ 0.05.

Among covariates, older age was significantly associated with lower perceived costs (*β* = −0.007, SE = 0.001, p < 0.001), with each additional year of age associated with approximately 0.7% lower perceived cost. Respondents without an established veterinarian perceived visit costs as substantially lower than those with one (*β* = −0.231, SE = 0.086, *p* = 0.007), corresponding to approximately 20.6% lower perceived cost. Household income was not a statistically significant predictor of perceived cost (β = 0.009, SE = 0.005, *p* = 0.068). Gender was not a significant predictor.

The interaction model (Model 2) revealed a statistically significant species-specific pattern. The Wald test confirmed that the two interaction terms were jointly significant (*F* (2, 2,212) = 3.13, *p* = 0.044), indicating that the association between community type and perceived cost differed between dog and cat owners; however, this *p*-value is at the conventional threshold and the interaction should be interpreted cautiously pending replication. For dogs, neither Suburban (*β* = −0.019, *p* = 0.742) nor Urban (β = 0.081, *p* = 0.223) differed significantly from Rural, indicating that dog owners’ cost perceptions were broadly similar regardless of community type. For cats, Suburban (*β* = 0.155, SE = 0.064, *p* = 0.015) and Urban (β = 0.139, SE = 0.068, *p* = 0.041) owners both perceived significantly higher routine visit costs than Rural cat owners. Combining the main community-type effect and the species interaction term, the total estimated Urban effect for cats was β = 0.220, corresponding to approximately 24.6% higher perceived costs relative to Rural cat owners.

## Discussion

In our sample, the results showed that the rural–urban cost perception gap is not a general phenomenon but rather is concentrated among cat owners. Because the study employs a cross-sectional design, these associations do not establish causal relationships. The proposed mechanisms are interpretive hypotheses consistent with the data and the broader literature. For dogs, perceived visit costs did not vary significantly by community type after controlling for covariates. For cats, however, rural owners perceived routine visit costs as meaningfully lower than their suburban and urban counterparts, with estimated marginal costs indicating that urban cat owners’ perceptions ($143.06) exceeded rural cat owners’ perceptions ($107.63) by approximately 25%. These findings advance our understanding of the behavioral mechanisms that may underlie the rural deficit in preventive veterinary care utilization, if these cost perceptions translate to the care-seeking patterns documented elsewhere in the veterinary utilization literature.

The AVMA (19) has long documented that the gap between the number of dog and cat visits to the veterinarian nationwide is not explained by lower cat ownership rates but by a persistent pattern of underutilization among cat owners. Our results suggest that the rural–urban cost perception gap may constitute one mechanism through which this feline care deficit operates in rural communities.

The absence of a rural–urban dog cost perception difference is informative. Dog owners report taking their pets to the veterinarian with greater regularity and are more likely to have an established veterinary relationship (14, 15). Perhaps frequent engagement with veterinary pricing creates experiential price signals that anchor expectations close to actual market rates. The null community-type effect for dogs thus offers an implicit control condition, suggesting that the cat-specific gap is not an artifact of broader rural information disadvantage but rather could reflect something particular about how rural cat ownership is practiced.

### Price anchoring and the rural fee environment

The most plausible explanation for the cat-specific rural perception gap could be price anchoring. Anchoring theory holds that consumers form price expectations from an initial reference point and adjust insufficiently from that reference point when new information becomes available (24–26). In the veterinary context, the primary source of price information is the veterinary visit itself. Rural cat owners, who by the utilization data use veterinary services far less frequently than urban cat owners (14, 19), accumulate fewer opportunities to update their price expectations from direct market experience. Those expectations remain anchored to older, potentially lower fee structures or to informal social network information about local pricing.

This mechanism is compounded by differences in the rural veterinary fee environment. Rural veterinary practices typically operate at lower price points than urban practices, reflecting lower overhead costs, reduced market competition, and local wage structures (12, 27). Spatial analysis confirms a significant income gradient for veterinarians, with earnings increasing with local practice density, such that rural practitioners earn less than their urban counterparts even after accounting for other factors (10). If rural cat owners do occasionally visit a veterinarian, they receive a price signal calibrated to rural market rates, potentially reinforcing an anchor below the national average visit cost of $202 reported for cats by the AVMA (14). Insufficient adjustment from this locally calibrated anchor would produce the pattern we observe: rural cat owners expect lower costs than urban cat owners, who are anchored to higher fee schedules. This mirrors evidence from human healthcare contexts that rural patients hold systematically different price expectations than urban patients, in part because rural provider fees differ structurally from urban fees (8, 28).

Beyond anchoring, our findings are consistent with theoretical accounts that emphasize cultural differences in how cats are kept and perceived across residential settings. In rural areas, cats are more commonly maintained as outdoor or working animals rather than indoor companions, a pattern that has been empirically linked to farm and village residence and to the role cats play in pest control (29). This utilitarian framing of rural cat ownership suppresses preventive care norms: owners who keep cats primarily as working animals rather than companions are less likely to seek routine veterinary care (15, 29). Cultural and sociodemographic factors have been widely documented as shaping pet ownership attitudes and care-seeking behavior (30), and the rural–urban axis represents one of the dimensions along which veterinary service utilization varies (15). Additionally, rural cat owners may not just anchor to lower prices but may expect to pay less because they seek a fundamentally different bundle of services. Where an urban cat owner expects an annual wellness exam, parasite prevention, and updated vaccinations, a rural owner may budget primarily for acute illness or injury treatment.

Several covariate findings merit discussion. The significant negative association between respondent age and perceived cost translates to approximately 0.7% lower perceived cost per additional year of age. The matches previous willingness-to-pay literature as Felsted (31) reports that respondents in the 50–69 age range have lower-than-average willingness to pay for many veterinary services. These findings are consistent with the hypothesis that older pet owners maintain price anchors formed during periods when veterinary fees were lower in real terms. Veterinary care costs have increased over the past two decades, outpacing general consumer price inflation by approximately 61% (32). Older owners who formed their price expectations before this cost inflation cycle will systematically underestimate current fees (26).

The veterinarian relationship effect has direct implications for outreach design: owners without an established veterinarian perceived costs roughly 21% lower than those with one, regardless of where they lived. Owners without a regular veterinary relationship have fewer opportunities to receive current price information through invoices, phone inquiries, or estimates. They may rely on secondhand sources for cost information, which may not reflect current prices. The Synchrony Lifetime of Care Study (33) found that nearly 80% of US pet owners underestimate their pet’s lifetime care costs. Neal and Greenberg (4) and Palmer et al. (22) document that geographic access barriers disproportionately affect rural residents, but the veterinarian relationship effect observed here was present across all three community types, suggesting it reflects a behavioral and relational dynamic that operates independently of geography.

Our findings should be interpreted in the context of a broader national pattern of veterinary cost underestimation. The 2025 Synchrony Lifetime of Care Study found that cat owners estimate lifetime care costs at under $6,000, far short of the study’s $20,000 minimum projection. In the study, almost 80% of pet owners across all species and geographies underestimate lifetime costs (33, 34). For individual visit costs, the AVMA (14) reports the average most-recent visit cost as $200 overall ($220 for dogs, $202 for cats). Yet this sample’s mean perceived costs ranged from $194–$243 for dogs and $144–$204 for cats, depending on community type. This is consistent with the pattern documented by Coe et al. (16), who identified perceived cost as a proximate determinant of care-seeking decisions, and with the finding that financial barriers affect 28% of pet-owning households (5). If pet owners routinely underestimate visit costs, the financial surprise at the point of care creates the conditions for acute care decisions to be made under stress rather than planned for in advance (35).

The welfare concern is not that rural cat owners expect low costs per se, but that if actual costs consistently exceed those expectations, as they would when rural owners encounter care outside their local fee environment or when pets develop conditions requiring more than a basic encounter. Financial unpreparedness may lead to declined or delayed care. Rural cat owners’ cost expectations, at a mean of $143.60, fall approximately 29% below the AVMA’s nationally reported average cat visit cost of $202. Even if rural feline care is genuinely less expensive, this magnitude of underestimation carries material welfare implications. A rural cat owner budgeting less than $100 for a routine visit would likely be unprepared for even a modestly complex presentation. This has direct implications for the preventive-to-acute care pipeline. Under preparation for routine costs increases the probability that owners will delay or forgo care until illness is advanced, at which point treatment costs escalate dramatically and outcomes deteriorate (6, 22). The Gallup-PetSmart survey (13) found that 52% of pet owners skipped recommended care in the prior year due to financial concerns, with the problem concentrated among those least financially prepared.

### Implications

If the issue is an information mismatch, the primary intervention point is pricing transparency. Rural cat owners need better information about current veterinary costs to plan appropriately. PetSmart Charities’ recent survey of veterinarians (36) found that 80% are unfamiliar with or infrequently apply the Spectrum of Care framework that would allow cost-adapted treatment options to be proactively offered, and that only 17% proactively address client financial concerns before making treatment recommendations. Proactive cost communication and pre-visit price disclosure would be a logical extension to veterinary practice, particularly for rural and underserved markets (6, 21).

If the cost charged by rural practices is significantly lower, the implication shifts toward supply-side interventions. Rural areas already face significant veterinary workforce shortages (11, 12), and veterinary care deserts affect an estimated 21 million US households (4). In areas where lower-cost rural practices exist but are geographically inaccessible, the rural underpricing perception may coexist with unmet need for veterinary services. The co-occurrence of low-cost expectations and low veterinary access in rural areas creates a particularly problematic dynamic: not only do rural cat owners not seek preventive care, but when they do encounter care outside their local area, they are disproportionately surprised by costs, consistent with Coe et al.’s (16) documentation of financial surprise as a driver of declined care recommendations.

The finding that having no established veterinarian independently depresses cost perceptions by 21% points to a specific, actionable intervention. Outreach programs that create initial veterinary contact, such as low-cost clinics, mobile veterinary services, and shelter medicine programs, may do two thing: provide direct care access while simultaneously calibrating pet owners’ price expectations through market exposure. Both of these outcomes align with evidence from King et al. (7)and Pasteur et al. (23) that perceived access to veterinary care improves significantly when community-based outreach programs create initial contact, even absent permanent access improvements. The ASPCA’s modeling of low-cost clinic market effects similarly finds that low-cost access points expand total market engagement without displacing full-cost practices (37), suggesting that cost calibration through low-cost contact need not threaten practice revenue.

Financial access solutions represent a complementary intervention. Payment plan availability has been shown to expand care-seeking behavior, with one national analysis estimating that approximately 8–9% of additional pet owners would seek routine veterinary care if financing options were accessible (17). The practice-level economics and risk profiles of clinic-backed payment plans suggest that well-structured financing programs are viable for most practice types and can be implemented without substantial default risk (38). Together, these findings argue for integrating payment flexibility with rural outreach as a dual strategy to address both the cost-perception gap and the structural affordability barrier.

Telehealth represents another avenue for bridging rural access gaps, with recent evidence suggesting that rural–urban differences in veterinary telehealth adoption are modest. In particular, rural pet owners are not substantially less likely to use digital veterinary services than their urban counterparts, indicating that technology-based access solutions may reach rural populations more effectively than geographic constraints alone would suggest (39). Combined with targeted cost-communication strategies, telehealth could serve as an entry point for calibrating rural cat owners’ price expectations through low-barrier virtual consultations before an in-person visit becomes necessary.

### Limitations

Several limitations of this study warrant acknowledgment. The primary outcome is self-reported perceived cost, which conflates what respondents expect to pay, recently paid, or consider fair; individual interpretations of “routine” care will also vary systematically, and rural owners anchoring to a narrower acute-care bundle may understate standard visit costs. Also, self-reported community type does not capture fine-grained variation in local veterinary market density and pricing within rural, suburban, and urban strata. Finally, actual cost data would be beneficial for comparison between rural and urban practices, but would require data that is not consistently tracked or collated across practices. Additional limitations include the following. First, the models control for age, income, gender, and established veterinarian status but omit potentially important confounders including education, race and ethnicity, pet insurance and wellness plan status, pet age and health status, prior veterinary utilization frequency, and local veterinary market characteristics. The observed R^2^ values of approximately 0.04–0.05 indicate that included covariates explain a small share of variance in perceived cost; unmeasured confounders could account for part of the observed community-type associations. Second, the survey was recruited through an online panel with quota sampling and post-stratification weighting, which does not guarantee probabilistic representativeness. Third, pet insurance and wellness plan status were not collected; insured owners may report perceived costs that reflect total invoice or out-of-pocket amounts differently depending on plan structure, introducing measurement heterogeneity we cannot fully resolve. Fourth, the study documents differences in cost perceptions but does not establish a direct link to care-seeking behavior, utilization rates, or animal welfare outcomes; the behavioral inferences in the Discussion draw on the broader literature and should be understood as interpretive hypotheses rather than demonstrated findings.

## Conclusion

This study provides national online sample evidence that rural–urban differences in perceived veterinary visit costs are species-specific. Among dog owners, cost perceptions did not vary meaningfully by community type, consistent with the view that regular veterinary engagement provides price signals sufficient to calibrate expectations regardless of geography. Among cat owners, rural residents perceived substantially lower visit costs than their suburban and urban counterparts, which is consistent with a price anchoring mechanism operating through lower local fee environments and reduced utilization-based price exposure. This finding extends the literature on rural veterinary access disparities (4, 22) by observing a cognitive mechanism that may compound structural barriers and contribute to the rural feline preventive care deficit. Two additional findings, that older owners and those without an established veterinarian hold substantially lower cost expectations, suggest actionable intervention points that could improve both financial preparedness and care continuity. Taken together, these results argue for integrating cost communication strategies with rural outreach programs, and for treating price transparency as a component of access, not merely a commercial concern.

## Data Availability

The raw data supporting the conclusions of this article will be made available by the authors, without undue reservation.
